# Biochemical Profiles, Mineral Accumulation, and Water-Soluble Fluoride Traits of 65 Tea (*Camellia sinensis*) Cultivars: For Germplasm Screening and Quality Evaluation

**DOI:** 10.3390/plants15091300

**Published:** 2026-04-23

**Authors:** Hongxiu Zhang, Lijin An, Danjuan Huang, Yangyang Sun, Lingyi Wang, Gaixiang Lei, Lirong Xu, Xun Chen

**Affiliations:** 1 Huanggang Academy of Agricultural Sciences, Huanggang 438000, China; 2 Key Laboratory of Tea Resources Comprehensive Utilization, Ministry of Agriculture and Rural Affairs, Institute of Fruit and Tea, Hubei Academy of Agricultural Sciences, Wuhan 430070, China; 3 Tea Industry Research Institute of Yingshan County, Huanggang 438000, China; 4 Chibi Agricultural and Rural Bureau, Chibi 437300, China

**Keywords:** tea varieties, biochemical constituents, cluster analysis, water-soluble fluoride, mineral elements, germplasm evaluation

## Abstract

The biochemical diversity among tea plant (*Camellia sinensis*) cultivars serves as the core material basis associated with tea quality and is of great significance for the innovation of tea germplasm resources and the genetic improvement of tea varieties. Here, we systematically analyzed 16 biochemical components, 7 mineral elements, and water-soluble fluoride (WSF) in 65 tea cultivars using multivariate analysis. These cultivars were grouped into high-component, high-epigallocatechin (EGC), low-component, and balanced-quality clusters. Significant variation was observed in quality-related parameters, including tea polyphenols, catechins, and amino acids and related quality indices. Mineral elements were significantly correlated with quality components, with potassium and boron showing significant correlation with the accumulation of these components. WSF content exhibited a pronounced cultivar-dependent variation, with more than 72% of cultivars containing less than 100 mg·kg^−1^. The balanced-quality cluster exhibited broad processing adaptability, making it suitable for producing various tea types. The high-EGC cluster is ideal for developing specialty functional teas. The high-component cluster offers core parental material for breeding cultivars high in tea polyphenols and epigallocatechin gallate. This study provides a scientific basis for the screening and utilization of tea germplasm resources and the development of new, high-quality, and safe tea varieties.

## 1. Introduction

Tea [*Camellia sinensis* (L.) O. Kuntze] is one of the three most widely consumed non-alcoholic beverages worldwide, providing substantial economic value and nutritional and health benefits [1]. Its quality and functional properties are primarily determined by its intrinsic biochemical composition. For example, tea polyphenols exhibit diverse bioactivities, including antibacterial, anti-inflammatory, and antioxidant effects [2]; caffeine exerts physiological functions such as central nervous system stimulation, diuresis, cardiac enhancement, and digestion promotion [3]; and free amino acids contribute to sedation, neuroprotection, and cognitive improvement [4]. Notably, epigallocatechin gallate (EGCG) is the predominant bioactive polyphenol in green tea and significantly influences its flavor profile [5]. Its content, however, varies considerably depending on cultivar genotype, harvest season, and processing method [6,7]. Beyond this sensory role, EGCG confers health benefits, including antitumor [8,9,10], antidiabetic [11], and antihypertensive effects [12], through various mechanisms.

Tea plants are typical fluoride-hyperaccumulating species. Under normal growing conditions, fluoride is primarily absorbed from soil and water [13]. In addition, in industrially polluted areas, atmospheric fluoride may also contribute to leaf accumulation [14,15]. Soil is the primary contributor to fluoride uptake, with water-soluble fluoride (WSF) being the main form absorbed by plants. Excessive fluoride accumulation has been shown to adversely affect plant growth, development, and tea quality. For example, Lu et al. reported significant correlations between leaf fluoride content and multiple quality-related parameters [16]. The extent of fluoride accumulation is largely governed by genetic variation among cultivars [17]. Marked differences in WSF content have been observed among the roots, stems, and leaves of different tea varieties [18]. In addition to their involvement in fluoride dynamics, mineral elements such as potassium [19], magnesium [20], and zinc [21] play essential roles as enzyme cofactors or structural components. These elements are widely involved in physiological processes, including photosynthesis [22], secondary metabolite biosynthesis [23], and ion translocation [24], which directly influence tea aroma, flavor, and overall quality [25]. Moreover, aluminum alleviates fluoride stress by promoting organic acid synthesis in tea plants, thereby mitigating adverse effects on physiological metabolism [26].

The genetic diversity of tea germplasm provides the foundation for germplasm conservation and cultivar improvement [27]. The diversity of biochemical constituents directly determines tea quality attributes [28], health benefits [29], and processing adaptability [30], and these biochemical components, therefore, serve as an important criterion for assessing germplasm value and guiding breeding programs [31]. Tea polyphenols, free amino acids, caffeine, and a range of aroma-related metabolites collectively form the chemical basis of tea flavor and functional properties [32]. By analyzing 114 tea germplasm resources, Luo et al. demonstrated that certain catechin components can serve as markers for cultivar discrimination and for evaluating suitability for oolong tea processing [33]. Biochemical trait–based evaluations of tea germplasm diversity have been applied in numerous studies, with important implications for discovering superior germplasm and breeding specialty cultivars [34,35]. For example, Kottawa-Arachchi et al. summarized biochemical and metabolite diversity in global tea germplasm but focused solely on primary and secondary metabolites, excluding safety-related parameters such as fluoride [7]. Wang et al. evaluated 47 tea accessions via entropy weight and grey relational methods, yet their analysis was limited to quality indicators and lacked nutritional and safety factors [36]. Consequently, existing studies largely focus on single metabolite classes or specific tea types, and a comprehensive, integrative assessment that jointly considers multiple quality indicators and safety and nutritional factors remains lacking.

In this study, 65 representative tea cultivars were selected for analysis. A systematic quantification of key quality-related biochemical components was conducted, including water-soluble extracts, tea polyphenols, free amino acids, caffeine, and major catechin monomers. The accumulation patterns of WSF and representative mineral elements were also profiled. This study aimed to: (1) comprehensively assess the biochemical diversity within the germplasm; (2) elucidate the relationships among the measured indicators; and (3) objectively classify cultivars based on their integrated biochemical profiles, using multivariate statistical approaches (correlation analysis, PCA, and HCA). The findings are expected to provide empirical support and a theoretical foundation for the scientific evaluation of tea germplasm resources, the construction of core germplasm collections, and the selection of parental lines for diverse breeding objectives.

## 2. Results

### 2.1. Descriptive Statistics of Major Biochemical Components in 65 Tea Plant Varieties

**Figure 1:**
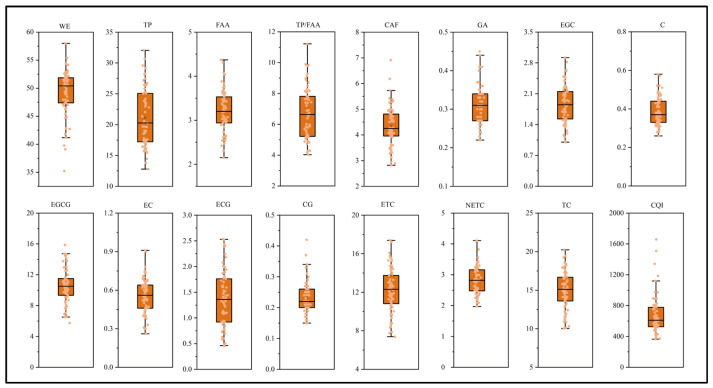
Variation and diversity of the biochemical components in 65 tea resources. WE: water extract; TP: tea polyphenols; FAA: free amino acids; CAF: caffeine; GA: gallic acid; EGC: epigallocatechin; C: catechin; EGCG: epigallocatechin gallate; EC: epicatechin; ECG: epicatechin gallate; CG: catechin gallate; ETC: esterified catechins; NETC: non-ester catechins; TC: total catechins; CQI: catechin quality index. Each colored dot represents the content of the corresponding sample.

The major biochemical constituents of tea leaves, such as catechins, caffeine, and tea polyphenols, are significantly influenced by the genetic background of the cultivar. WE content was the highest among all measured biochemical components, with the cultivar Bixiangzao at 58% and Bayu Tezao at 35.2%; 95% of the accessions exhibited WE content of 40–58%. TP content ranged from 12.8% to 32.1%, while FAA content ranged from 2.2% to 4.4%, and CAF content ranged from 2.8 to 6.9 mg·g^−1^ dry weight (DW). The ratio of tea polyphenols to free amino acids (TP/FAA), a key indicator of tea quality and processing suitability, ranged from 4.0 to 11.2, with 16 accessions exhibiting a ratio exceeding 8. These results suggest that while the majority of the tested cultivars are suitable for green tea production, those with higher TP/FAA ratios may also be favorable for black tea processing (Figure 1).

Analysis of catechin components in the tea samples indicated that EGCG is the predominant catechin and a key determinant of tea quality. EGCG content varied broadly from 5.7 to 15.9 mg·g^−1^ DW, with a mean of 10.6 ± 2.3 mg·g^−1^ DW. Notably, among the 65 tea germplasm accessions, 41 (63.1%) exhibited EGCG levels above 10 mg·g^−1^ DW. The remaining catechin components showed the following ranges: gallic acid (GA) 0.2–0.5 mg·g^−1^ DW; EGC 1.0–2.9 mg·g^−1^ DW; C 0.3–0.6 mg·g^−1^ DW; EC 0.3–0.9 mg·g^−1^ DW; ECG 0.5–2.5 mg·g^−1^ DW; CG 0.2–0.4 mg·g^−1^ DW. Collectively, these variations reflect diverse catechin metabolism patterns and underpin the quality potential across different tea cultivars.

The CV is a key metric for quantifying phenotypic variability in germplasm resources based on biochemical traits. The lowest CV was observed for WE content (8.2%), while the highest CV was observed for ECG (40.3%). Among the catechin components, all CVs exceeded 18.5%, indicating substantial phenotypic variation and rich biochemical diversity in catechin composition. Collectively, these results suggest that the phenotypic variation range of WE content is relatively narrow under the present experimental conditions. However, the large phenotypic variation in catechin-related traits provides abundant potential for germplasm screening and breeding utilization. Furthermore, the *H′* diversity index across the 16 biochemical indices exceeded 1.7, underscoring the rich biochemical diversity present among the 65 tea accessions (Table 1).

### 2.2. Correlation Analysis of Biochemical Components

**Figure 2:**
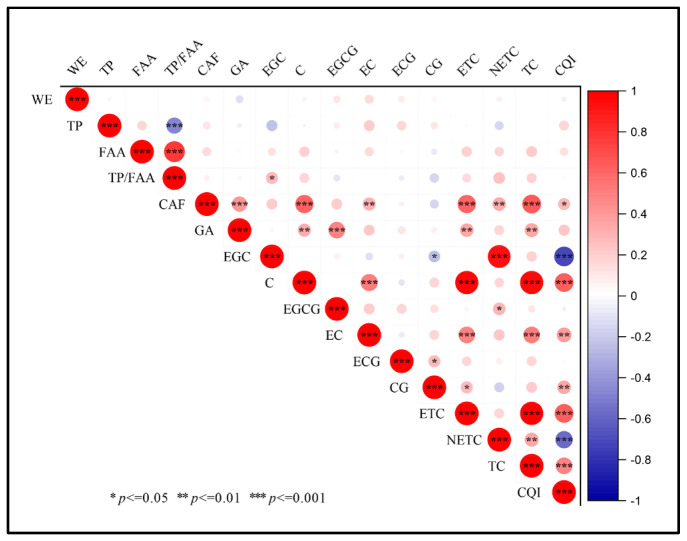
Correlation heatmap displaying Pearson correlation coefficients between biochemical components across the 65 tea varieties. Positive and negative correlations are depicted in red and blue, respectively, with darker shades indicating stronger correlations. Correlations marked with *, **, and *** indicate statistical significance at the *p* ≤ 0.05, 0.01, and 0.001 levels, respectively.

A correlation analysis was conducted on 16 quality-related chemical components and indicators across the 65 tea plant varieties. CAF showed significant positive correlations with GA, C, EC, ETC, NETC, TC, and CQI (*p* < 0.05), suggesting synergistic regulation among their metabolic pathways. GA correlated positively with C, EGCG, ETC, and TC (*p* < 0.01). Only EGC and CG exhibited weak negative correlations with CQI, with overall associations among the tested indices being predominantly positive (Figure 2).

### 2.3. Cluster Analysis of 65 Tea Plant Varieties

**Figure 3:**
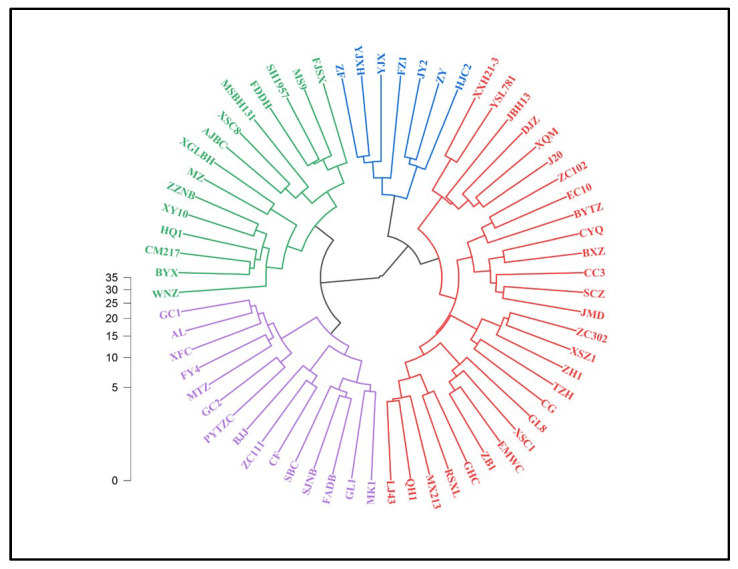
Dendrogram of hierarchical cluster analysis for 65 cultivars based on biochemical components. At a Euclidean distance threshold of 14.0, the 65 tea accessions were classified into four clusters, as indicated by different colors. All abbreviations are detailed in Supplementary Table S1.

To reduce redundancy among the variables, correlation analysis was first performed, followed by HCA using Ward’s method with squared Euclidean distance as the distance metric. The 65 tea accessions were partitioned into four distinct clusters at a squared Euclidean distance cutoff of 14.0 (Figure 3). Cluster I comprised seven accessions, including Zifeng, Yujinxiang, and Yujinxiang. Cluster II was the largest, containing 28 accessions, such as Zhongcha 302, Tezaohuang, and Shuchazao. Cluster III consisted of 15 accessions, including Guilü 1, Pingyang Tezaocha, and Zhongcha 111. Cluster IV contained 16 accessions, including Xingyang 10, Wuniuzao, and Xishancha 8.

All biochemical components exhibited significant inter-cluster variation (*p* < 0.05), with the exception of WE, which demonstrated no significant differences among clusters. Cluster I showed the significantly highest mean values for multiple core quality indicators (labeled with letter a, *p* < 0.05). Specifically, TP averaged 24.2 ± 5.6%, FAA averaged 3.4 ± 0.3%, and EGCG averaged 13.6 ± 1.5 mg·g^−1^ DW. This cluster exhibited the highest average levels of ETC and TC (15.6 ± 1.3 mg·g^−1^ and 18.0 ± 1.2 mg·g^−1^, respectively). CQI was 1179.1 ± 304.3, a substantially higher value than that obtained for the other clusters. Most components within this cluster exhibited low CVs, suggesting relatively stable trait expression (Table 2).

Cluster II was primarily characterized by the significantly highest contents of EGC (2.2 ± 0.3 mg·g^−1^) and NETC (3.2 ± 0.3 mg·g^−1^) among all clusters (*p* < 0.05). This cluster also exhibited the relatively high TP/FAA ratio (6.5 ± 1.5), reflecting greater intra-cluster variability in components such as FAA and EC. In contrast, Cluster III exhibited the significantly lowest mean values for most biochemical components (*p* < 0.05). Notable examples included CAF (3.9 ± 0.5 mg·g^−1^ DW), EGCG (9.2 ± 1.7 mg·g^−1^ DW), and TC (13.43 ± 2.0 mg·g^−1^ DW), together with a relatively low TP/FAA ratio (6.7 ± 1.8). Certain constituents, including EC and ECG, exhibited significant intra-cluster variation. Cluster IV exhibited intermediate mean values for most biochemical parameters. However, its CQI was high (717.9 ± 175.7), ranking second only to Cluster I. Furthermore, this cluster exhibited high component stability, with the lowest or second-lowest coefficients of variation among the four clusters for CAF, GA, and C (Table 2).

### 2.4. Principal Component Analysis Based on Clustering Results and Spectral Features

To reduce dimensionality and help interpret the compositional structure, PCA was performed on 11 biochemical parameters measured in the 65 tea accessions (Table 3). The first five principal components (PCs), with eigenvalues greater than 1 and individual contributions exceeding 5.0% of the total variance, were retained, which collectively explained 69.7% of the total variance. PC1 explained 22.2% of the total variance and was predominantly associated with EGCG content, with caffeine as a secondary contributor. PC2 accounted for 14.2%, with C content as the major loading. PC3 explained 12.7% and was primarily driven by EC content. PC4 accounted for 10.8% of the variance and was largely driven by FAA. PC5 contributed 9.7% and was mainly associated with ECG content.

**Figure 4:**
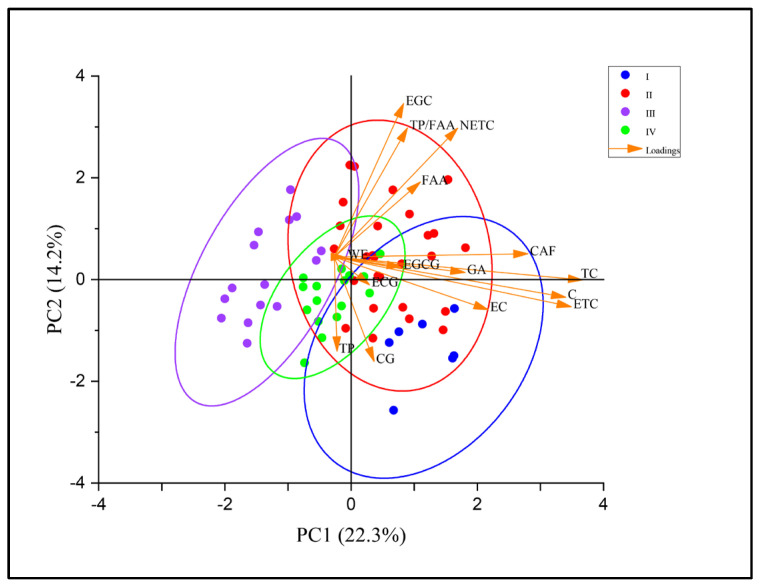
Principal component analysis plot showing the distribution of the four clusters. Different colored dots represent distinct clusters; arrows indicate loading vectors of biochemical indicators; PC1 and PC2 represent the first and second principal components, respectively.

To validate the robustness of the four-cluster classification derived from hierarchical clustering, PCA was performed on the same biochemical dataset, and the 65 accessions were projected onto the PC1–PC2 score plane (Figure 4). The plot revealed clear spatial separation among the four clusters. Cluster I is located exclusively in the positive PC1 region, consistent with its biochemical signature of high TP and EC contents, which load positively on PC1. Cluster II was distinctly separated along the positive PC2 axis, which directly confirms its unique feature of significantly higher EGC content than the other clusters. Clusters III and IV predominantly occupy the negative PC1 region and are centered on the PC2 axis, corresponding to comparatively low polyphenol levels and relatively balanced TP/FAA ratios. Overall, the PCA results corroborate the four-cluster structure and align with the biochemical profiles characterizing each cluster.

### 2.5. Water-Soluble Fluoride Content in 65 Varieties

**Figure 5:**
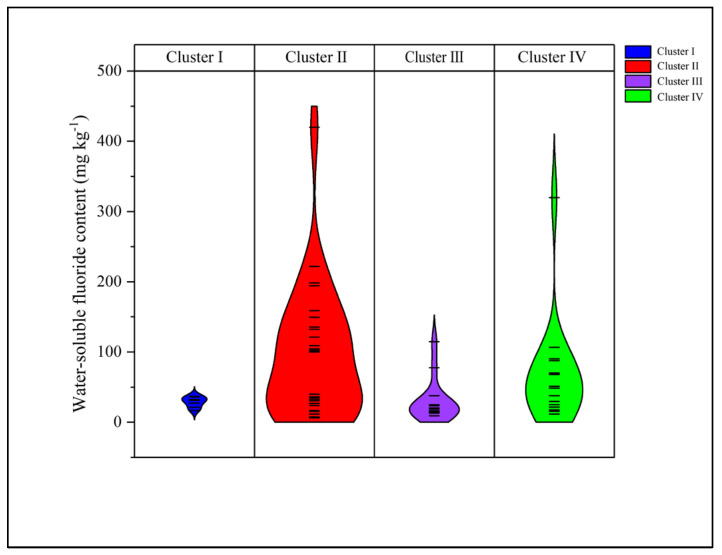
Violin plot of cumulative distribution and probability density of water-soluble fluoride content (mg·kg^−1^) among 65 tea varieties across Clusters I–IV. The width of each violin corresponds to the kernel density of data at different values.

WSF content was systematically determined across the 65 tea accessions. Differential analysis was then conducted on the clustering results to evaluate the safety profiles and cluster-specific characteristics of fluoride accumulation. The WSF content among the 65 accessions ranged from 6.3 to 419.9 mg·kg^−1^, with a mean value of 64.5 ± 73.9 mg·kg^−1^ (Figure 5). The four clusters exhibited distinct patterns of fluoride accumulation. Cluster I exhibited consistently low fluoride levels, with values ranging from 16.5 to 36.7 mg·kg^−1^ and a mean of 28.7 ± 7.6 mg·kg^−1^. The CV of 26.4% was the lowest among the four clusters, indicating the greatest intra-cluster uniformity in fluoride content. Cluster II displayed the widest range of fluoride accumulation, ranging from 6.3 to 419.9 mg·kg^−1^, with a mean of 94.5 ± 92.4 mg·kg^−1^ and a CV of 97.9%, indicating substantial variability within this cluster. This cluster encompassed both high-fluoride accessions, such as Xiaoqingmiao (419.9 mg·kg^−1^) and Echa 10 (221.7 mg·kg^−1^), and low-fluoride accessions, including Shuchazao (6.3 mg·kg^−1^). Cluster III was characterized by low to moderate fluoride levels, ranging from 9.3 to 114.7 mg·kg^−1^, with a mean of 29.9 ± 29.8 mg·kg^−1^ and a CV of 99.4%. All accessions in this cluster exhibited WSF concentrations below 50 mg·kg^−1^, with the exception of Mingke 1 (114.7 mg·kg^−1^) and Fuyun 4 (77.5 mg·kg^−1^). Cluster IV showed moderate to high fluoride accumulation, with values ranging from 11.5 to 319.8 mg·kg^−1^ and a mean of 66.8 ± 71.3 mg·kg^−1^ and a CV of 106.6%, representing the highest level of within-cluster variation (Figure 5).

In the study, a weak correlation was observed between WSF content and clustering based on biochemical components, suggesting that fluoride accumulation is a genetic trait independent of major biochemical quality traits. Although previous studies have highlighted the importance of environmental factors in fluoride accumulation, the substantial variation in WSF content among cultivars within the same biochemical cluster indicates that genetic characteristics also significantly influence this process. This intra-cluster variation reflects physiological differences in fluoride uptake among cultivars, providing a genetic basis for the targeted selection of low-fluoride or high-fluoride germplasm.

### 2.6. Mineral Element Content in Representative Tea Plant Varieties

A subset of 17 representative accessions was selected from the 65 tea cultivars based on the results of the clustering and principal component analyses. Among the analyzed elements, K exhibited the highest mean concentration (22,331.43 ± 1613.5 mg·kg^−1^) and the lowest CV (7.2%), indicating relatively uniform accumulation across the selected varieties. The mean concentrations of Ca, Al, and Mn were 1158.92, 521.66, and 2828.57 mg·kg^−1^, respectively, with coefficients of variation ranging from 32.0% to 34.5%. In contrast, B and Zn exhibited the greatest varietal variability, with CVs of 57.5% and 55.2%, respectively (Table 4).

**Figure 6:**
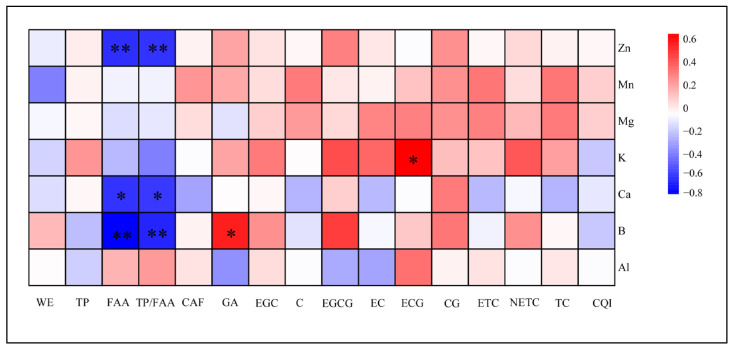
Correlation analysis between mineral element content and quality component indicators of 17 varieties. Heatmap showing Pearson correlation coefficients between mineral element contents and quality component indicators across 17 tea varieties. Positive and negative correlations are represented in red and blue, respectively, with darker shades indicating stronger correlations. * and ** denote significant (*p* ≤ 0.05) and highly significant (*p* < 0.01) results, respectively.

To explore the associations between mineral elements and key biochemical indicators related to tea quality, Pearson correlation analysis was performed between the concentrations of seven mineral elements and key biochemical quality indicators (Figure 6). B, Zn, and Ca contents were significantly negatively correlated with FAA content, with B showing the strongest association (*r* = −0.747), followed by Zn (*r* = −0.609) and Ca (*r* = −0.598). In contrast, these three elements showed negative correlations with the TP/FAA ratio, indicating that higher levels of these minerals co-occur with lower TP/FAA ratios. Additionally, K content showed a positive correlation with ECG (*r* = 0.641), while B content was positively correlated with GA (*r* = 0.553). The contents of the remaining mineral elements did not show significant correlations with any of the measured biochemical components.

## 3. Discussion

### 3.1. Biochemical Diversity and Taxonomic Characteristics of Tea Plants

The genetic diversity of tea germplasm resources constitutes a critical foundation for tea genetic improvement [37]. Systematic biochemical characterization of germplasm resources is an effective approach for revealing intrinsic biochemical variation and evaluating their breeding potential [38]. In this study, WE content exhibited the lowest CV, indicating high phenotypic stability for this trait within the tested population and relatively limited phenotypic variation available for phenotypic selection (Table 1). This finding is consistent with previous reports [39] and may reflect the composite nature of primary metabolic products, which are governed by polygenic regulatory networks and characterized by evolutionary conservatism. In contrast, catechin constituents exhibit substantial variation in content across cultivars. These compounds are the core determinants of tea flavor quality and physiological activity [40]. This pronounced variability mirrors the metabolic plasticity of tea plants during adaptation to diverse ecological environments. From a breeding perspective, a large CV indicates broader potential for phenotypic improvement of these traits, suggesting greater responsiveness to selection in quality-directed breeding and cultivar development [41].

The biochemical clustering of tea germplasm reveals the underlying biochemical diversity among the accessions and translates this variation into a classification framework with direct breeding implications. Distinct catechin profiles emerged across the four clusters. Cluster I, characterized by high levels of EC, represents a promising source of parental material for breeding high-functionality tea products. Cluster II, distinguished by elevated EGC and NETC contents, offers a genetic resource for developing specialty teas with targeted health benefits. In contrast, Clusters III and IV exhibit stable biochemical profiles and are suitable as backbone parents for high-quality green tea breeding programs (Table 2). This metabolically informed classification framework provides practical guidance for parental selection and germplasm utilization tailored to diverse breeding objectives. By enabling more targeted crosses, this framework can potentially enhance breeding efficiency, shorten selection cycles, and accelerate the development of novel, high-quality specialty tea cultivars [42].

### 3.2. Relationship Between Mineral Element Accumulation, Quality, and Fluoride Accumulation

This study systematically quantified the mineral element concentrations in 17 tea accessions, which were stratified and selected across the four clusters derived from biochemical clustering of the 65 germplasms, and examined their associations with biochemical groupings and quality-related traits. The correlation analysis (Figure 6) revealed that K and B were significantly correlated with multiple core quality indicators, and these significant phenotypic associations were noted in relation to carbon and nitrogen partitioning and secondary metabolite biosynthetic pathways [43,44]. Additionally, WSF content was measured across all 65 tea accessions, consistent with the view that fluoride accumulation is a prominent cultivar-dependent trait in tea plants. This finding is consistent with the large-scale study of 85 cultivars by Wen et al. [45]. Importantly, because all plant materials in the present study were cultivated in the same plot under uniform soil fluoride conditions and identical management practices, the observed inter-cultivar differences in WSF content mainly reflect distinct genotypic variation in fluoride accumulation capacity rather than environmental effects. Consistent with this interpretation, Ruan et al. [20] documented obvious genotypic differences in fluoride accumulation among tea varieties. Furthermore, Yang et al. [46] demonstrated at both physiological and molecular levels that fluoride-tolerant and fluoride-sensitive varieties employ distinct adaptive mechanisms in response to fluoride stress, underpinned by reprogramming of energy metabolism and differential regulation of stress-responsive gene expression.

Notably, no significant differences in WSF content were detected among the four biochemical clusters, with over 72% of the accessions exhibiting levels below 100 mg·kg^−1^ (Figure 5). Previous studies have established that fluoride preferentially accumulates in mature leaves, with cell walls serving as the primary accumulation site [47]. Given that the samples analyzed in the present study consisted of young shoots (one bud and two leaves) and that fluoride translocation from older to younger leaves is limited in tea plants, the measured fluoride concentrations in young tissues may not fully reflect the inherent accumulation capacity of individual cultivars. This tissue-specific effect may partly explain why marked inter-cluster differences were not observed in this study. These findings underscore that the selection of tissue, particularly the developmental stage of leaves, is crucial to evaluating fluoride accumulation in tea. Consequently, future research should systematically compare fluoride concentrations across various leaf developmental stages to thoroughly elucidate the accumulation patterns specific to different cultivars.

## 4. Materials and Methods

### 4.1. Materials

All 65 accessions of *Camellia sinensis* were collected from the Ten-Thousand-Mu Tea Germplasm Resource Nursery in Chibi City, Hubei Province, China (229°36′30.15″ N, 113°45′20.78″ E). All expsoil type, water, fertilizer, and pest control measures. One bud and two leaves shoots were uniformly collected in late August (Summer and Fall tea season) in 2025. All samples were immediately fixed at 120 °C for 2 min and dried at 65 °C to constant weight. After sieving through a 40-mesh standard sieve, samples were sealed in lightproof containers and stored at −20 °C for subsequent analysis (Table 5).

### 4.2. Biochemical Component Analysis

Total tea polyphenol (TP) content and catechin profiles were quantified following the protocols GB/T 8313-2018 [48], GB/T 8312-2013 [49]. Briefly, 0.2 g of dried tea powder was extracted using 8.0 mL of 70% methanol (*v*/*v*) at 70 °C for 20 min. After centrifugation (12,000× *g*, 10 min), the supernatant was collected and adjusted to a final volume of 10.0 mL. The extract was subsequently analyzed by high-performance liquid chromatography (HPLC) using an Agilent ZORBAX SB-C18 analytical column (250 mm × 4.6 mm, 5 μm; Agilent Technologies, Santa Clara, CA, USA) equipped with a matching guard column. The mobile phase consisted of 0.2% acetic acid (A) and acetonitrile (B). Chromatographic conditions were set as follows: detection wavelength 280 nm, flow rate 1 mL/min, column temperature 40 °C, and injection volume 10 μL. Gradient elution was performed as follows: 0–4 min, 93.5% A and 6.5% B; 4–16 min, 85% A and 15% B; 16–28 min, 75% A and 25% B; 28–35 min, 93.5% A and 6.5% B. Individual catechins including epigallocatechin (EGC), epicatechin (EC), catechin (C), epigallocatechin gallate (EGCG), epicatechin gallate (ECG), catechin gallate (CG), and caffeine (CAF) were identified by comparison with authentic standards (purity ≥ 98%; Sigma-Aldrich, St. Louis, MO, USA). All samples were subjected to three technical replicates, with the results reported as the mean.

In accordance with GB/T 8305-2013 [50] and GB/T 8314-2013 [51], we determined the levels of water extract (WE) and free amino acids (FAA). The extraction involved boiling 2.0 g of tea powder in 300 mL of boiling water for 45 min (shaken every 10 min). The resulting solution was filtered, brought to a final volume of 500 mL, and used for subsequent analyses. WE were calculated by the difference method, involving drying and weighing the filter residue. It should be noted that catechin profiling was carried out on the methanol extract prepared for total polyphenol analysis, distinct from this aqueous extract. All samples were analyzed with three technical replicates to ensure the accuracy and repeatability of the analytical results.

### 4.3. Determination of Water-Soluble Fluoride Content

Following Wang et al. [52], WSF content was determined by extracting 0.20 g of tea powder in 40 mL of boiling water for 15 min. The mixture was cooled, and 10 mL of the extract was mixed with 10 mL of TISAB. The potential (mV) was recorded with a fluoride ion-selective electrode (PerfectION™; Mettler Toledo, Columbus, OH, USA), total TISAB was added to eliminate matrix interference. Quantification was carried out using the standard curve method, with blank controls and parallel samples included for quality control. To prepare the TISAB buffer, 58 g of sodium chloride (NaCl), 120 g of trisodium citrate dihydrate (C_6_H_5_Na_3_O_7_·2H_2_O), and 57 mL of glacial acetic acid were dissolved in approximately 700 mL of deionized water. The pH was adjusted to 5.0–5.2 with 10 mol·L^−1^ sodium hydroxide (NaOH), and the solution was topped up to 1 L.

### 4.4. Determination of Mineral Element Content

The contents of potassium (K), boron (B), zinc (Zn), calcium (Ca), manganese (Mn), aluminum (Al), and magnesium (Mg) were determined in tea leaf samples by inductively coupled plasma mass spectrometry (ICP-MS; Agilent 7900, Agilent Technologies, Santa Clara, CA, USA) as described by Liu et al. [53]. For sample preparation, 0.2 g of tea powder was mixed with 5 mL of concentrated nitric acid (HNO_3_) and 2 mL of perchloric acid (HClO_4_) and pre-digested for 12 h. Microwave digestion was performed with a three-step program: increasing to 120 °C (5 min), ramping to 180 °C (15 min), and finally ramping to 200 °C (20 min). The cooled solution was transferred to a 50 mL volumetric flask, made up to volume with ultrapure water, and filtered through a 0.45 μm membrane. Standard curve preparation: Mineral element standard stock solutions (National Center of Analysis and Testing for Nonferrous Metals and Electronic Materials, Beijing, China) were serially diluted with 1% dilute nitric acid to different concentration gradients. The mixed elements (Zn, Mn, Al, B, and Mg) were diluted to 0, 0.01, 0.05, 0.5, 1.0, 5, and 10 μg/mL; Ca was diluted to 0, 0.05, 0.25, 2.5, 5.0, 25, and 50 μg/mL; K was diluted to 0, 1, 5, 10, and 20 μg/mL, and the standard curve was automatically generated by the instrument. The good linearity of the analysis curve was *r*^2^ > 0.999. All samples were analyzed in three technical replicates to ensure data accuracy and reliability. Instrument parameters included an RF power of 1550 W, a coaxial nebulizer, a sampling depth of 9.5 mm, and a peristaltic pump speed of 0.3 rpm. Sample flow rate: 0.4 mL min^−1^. The carrier and gas-compensation flow rates were set to 0.85 L min^−1^ and 0.15 L min^−1^.

### 4.5. Multivariate Statistical Analysis

Biochemical indices, including total catechins (TC), non-esterified catechins (NETC), esterified catechins (ETC), and catechin quality index (CQI), followed these formulas: TC = EGC + EC + C + EGCG + ECG + CG; NETC = EGC + EC + C; ETC = EGCG + ECG + CG; CQI = [(EGCG + ECG)/EGC] × 100. Using WPS Office software, we determined the mean, standard deviation (SD), and coefficient of variation (CV). The CV was derived by dividing the SD by the mean and multiplying by 100. Furthermore, the Shannon–Wiener diversity index (*H′*) was calculated as *H′* = −ΣPj ln Pj, with Pj denoting the frequency of the jth coded value for each trait.

Pearson correlation analysis was performed to examine the linear relationships among biochemical components and between mineral elements and quality indicators. *p*-values were calculated to indicate the statistical significance of correlations. HCA was performed using Ward’s method, with squared Euclidean distance used as the similarity measure. The optimal number of clusters was determined by a fixed distance threshold derived from the dendrogram topology with cross-validation using two independent software packages, and a dendrogram was constructed to delineate the grouping of tea accessions. PCA was performed on the biochemical indicators using SPSS 26.0 (IBM Corp., Armonk, NY, USA; https://www.ibm.com/products/spss-statistics, accessed on 3 January 2026) to extract principal components and their corresponding contribution rates. PCA score plots and loading plots were generated using Origin 2024 (OriginLab Corp., Northampton, MA, USA; https://www.originlab.com/) to visualize the distribution of accessions in the principal component space and identify the contribution of each biochemical indicator to the principal components, thereby revealing the key variables driving the classification.

## 5. Conclusions

This study systematically profiled the biochemical composition of 65 *Camellia sinensis* accessions, revealing substantial phenotypic diversity within the tea germplasm. HCA partitioned the accessions into four distinct groups with marked variation in catechin profiles. Esterified and non-esterified catechins displayed largely independent accumulation patterns across the germplasm. Correlation analysis revealed significant associations between mineral elements and key quality-related traits; in particular, K and B showed significant positive correlation with the contents of major biochemical constituents. WSF content showed pronounced cultivar-dependent variation, with over 72% of accessions accumulating less than 100 mg·kg^−1^. The wide range of inter-cultivar differences in fluoride accumulation demonstrates substantial genotypic variation. Nevertheless, this study has several limitations. First, as all accessions were grown in a common environment with uniform management, genotype–environment interactions could not be evaluated. Second, soil fluoride content was not determined; future studies should integrate soil and tea leaf fluoride data to better understand fluoride accumulation patterns. Third, the observed correlations between mineral elements and biochemical components are merely associative, and the underlying molecular and metabolic regulatory mechanisms await further investigation. Finally, this study only measured water-soluble fluoride, without analyzing the accumulation profiles of different fluoride fractions in tea leaves. Collectively, the integrated, multicriteria evaluation framework developed in this study provides a theoretical basis and practical foundation for tea breeding programs aimed at simultaneously improving quality and ensuring consumer safety in new cultivars.

## Figures and Tables

**Table 1:** Basic statistical parameters of main biochemical components.

Biochemical Component	Maximum	Minimum	Mean ± Standard Deviation	Coefficient of Variation	Diversity Index
WE%	58.00	35.22	49.26 ± 4.07	8.27	1.84
TP%	32.05	12.79	21.15 ± 4.70	22.23	1.84
FAA%	4.37	2.15	3.20 ± 0.48	14.97	1.83
TP/FAA	11.22	4.03	6.72 ± 1.67	24.90	1.77
CAF mg·g^−1^	6.91	2.82	4.35 ± 0.78	17.97	1.84
GA mg·g^−1^	0.45	0.22	0.31 ± 0.05	16.62	1.80
EGC mg·g^−1^	2.92	1.00	1.86 ± 0.43	23.21	1.84
C mg·g^−1^	0.58	0.26	0.39 ± 0.07	18.46	1.81
EGCG mg·g^−1^	15.87	5.74	10.55 ± 2.29	21.73	1.83
EC mg·g^−1^	0.91	0.26	0.60 ± 0.14	25.11	1.82
ECG mg·g^−1^	2.53	0.46	1.39 ± 0.56	40.32	1.72
CG mg·g^−1^	0.42	0.15	0.23 ± 0.05	21.58	1.78
ETC mg·g^−1^	17.38	7.39	12.17 ± 2.33	19.12	1.84
NETC mg·g^−1^	4.11	1.97	2.81 ± 0.46	16.25	1.84
TC mg·g^−1^	20.23	10.02	14.99 ± 2.44	16.31	1.84
CQI	1658.43	364.91	678.74 ± 250.29	36.87	1.84

**Table 2:** Comparison of statistical parameters of quality chemical components among different clusters.

No	Main Quality Components	Cluster I		Cluster II		Cluster III		Cluster IV	
		Mean ± SD	CV%	Mean ± SD	CV%	Mean ± SD	CV%	Mean ± SD	CV%
1	WE%	49.44 ± 3.27 ^a^	6.62	49.52 ± 3.81 ^a^	9.21	49.18 ± 1.42 ^a^	6.45	49.47 ± 3.88 ^a^	7.84
2	TP%	24.23 ± 5.64 ^a^	23.66	21.78 ± 4.34 ^b^	18.78	20.16 ± 4.37 ^bc^	20.10	18.64 ± 3.71 ^c^	19.93
3	FAA%	3.40 ± 0.25 ^a^	7.41	3.40 ± 0.53 ^a^	17.37	3.06 ± 0.42 ^b^	15.87	3.10 ± 0.34 ^b^	11.05
4	TP/FAA	7.12 ± 1.42 ^a^	20.02	6.53 ± 1.46 ^b^	24.39	6.71 ± 1.78a ^b^	25.68	6.08 ± 1.28 ^c^	21.01
5	CAF mg·g^−1^	4.73 ± 0.89 ^a^	18.85	4.84 ± 0.69 ^a^	15.72	3.94 ± 0.54 ^c^	13.17	4.16 ± 0.36 ^b^	8.73
6	GA mg·g^−1^	0.35 ± 0.03 ^a^	8.16	0.33 ± 0.04 ^b^	16.39	0.29 ± 0.05 ^c^	20.79	0.29 ± 0.03 ^c^	9.71
7	EGC mg·g^−1^	1.36 ± 0.33 ^c^	24.31	2.12 ± 0.34 ^a^	15.43	1.79 ± 0.39 ^ab^	19.10	1.69 ± 0.35 ^b^	20.81
8	C mg·g^−1^	0.40 ± 0.06 ^a^	10.98	0.41 ± 0.06 ^a^	14.00	0.37 ± 0.07 ^b^	12.98	0.37 ± 0.08 ^b^	9.43
9	EGCG mg·g^−1^	13.63 ± 1.50 ^a^	14.94	11.62 ± 1.72 ^b^	13.20	9.22 ± 1.71 ^c^	23.60	10.43 ± 0.98 ^c^	22.66
10	EC mg·g^−1^	0.67 ± 0.04 ^a^	5.74	0.65 ± 0.13 ^a^	26.11	0.49 ± 0.11 ^c^	23.35	0.56 ± 0.09 ^b^	16.05
11	ECG mg·g^−1^	1.55 ± 0.79 ^a^	50.74	1.40 ± 0.6 ^ab^	42.32	1.33 ± 0.50 ^b^	32.89	1.37 ± 0.47 ^b^	34.47
12	CG mg·g^−1^	0.30 ± 0.08 ^a^	25.38	0.22 ± 0.04 ^b^	17.72	0.22 ± 0.04 ^b^	14.28	0.23 ± 0.04 ^b^	17.09
13	ETC mg·g^−1^	15.58 ± 1.27 ^a^	8.16	13.25 ± 1.51 ^b^	10.90	10.77 ± 1.86 ^c^	10.22	12.03 ± 0.99 ^bc^	8.25
14	NETC mg·g^−1^	2.43 ± 0.33 ^c^	13.51	3.17 ± 0.34 ^a^	10.21	2.65 ± 0.38 ^b^	13.71	2.63 ± 0.39 ^b^	14.98
15	TC mg·g^−1^	17.95 ± 1.17 ^a^	6.54	16.43 ± 1.52 ^b^	8.98	13.43 ± 2.02 ^c^	8.95	14.69 ± 1.06 ^c^	7.20
16	CQI	1179.09 ± 304.27 ^a^	25.81	629.70 ± 127.77 ^b^	21.14	607.34 ± 162.55 ^b^	18.59	717.96 ± 175.74 ^b^	24.48

Note: Different lowercase letters (a, b, c) in the same row indicate significant differences (*p* < 0.05) among the four clusters, as determined by one-way ANOVA followed by Duncan’s multiple range test.

**Table 3:** Principal component analysis of biochemical components in 65 tea tree cultivar resources.

Biochemical Components	PC1	PC2	PC3	PC4	PC5
WT%	0.001	−0.028	−0.036	−0.049	0.004
FAA%	0.008	−0.036	−0.149	0.813	0.117
TP%	−0.105	0.027	0.048	−0.03	−0.043
CAF mg·g^−1^	0.462	−0.052	−0.322	0.129	0.185
GA mg·g^−1^	0.046	0.509	0.023	−0.098	−0.055
EGC mg·g^−1^	0.057	−0.019	−0.365	−0.338	0.332
EGCG mg·g^−1^	0.51	−0.179	0.156	−0.152	0.004
C mg·g^−1^	−0.208	0.67	−0.014	0.046	−0.063
EC mg·g^−1^	0.237	0.081	0.159	0.134	−0.266
ECG mg·g^−1^	0.011	−0.049	0.071	0.071	0.783
Variance contribution rate	22.235	14.240	12.710	10.762	9.728
Cumulative contribution rate	22.235	36.475	49.185	59.947	69.675

**Table 4:** Variation in the content of seven mineral elements in 17 tea tree cultivar resources.

Mineral Element (mg·kg^−1^)	Maximum	Minimum	Mean ± SD	CV%
Ca	1821.50	666.46	1158.92 ± 371.24	32.03
Mg	2600.56	1532.41	2139.87 ± 364.76	17.05
K	25,014.64	19,453.30	22,331.43 ± 1613.46	7.23
Al	929.92	250.20	521.66 ± 169.05	32.41
B	89.28	19.83	41.46 ± 23.85	57.52
Mn	5071.32	1245.38	2828.57 ± 975.41	34.48
Zn	67.03	10.22	31.12 ± 17.16	55.15

**Table 5:** Names and sources of tested tea varieties.

Number	Species Name	Source	Number	Species Name	Source	Number	Species Name	Source
1	Longjing 43	Zhejiang	23	Zhenzhun Nai Bai	Zhejiang	45	Yabukita	Japan
2	Fujian Shuixian	Fujian	24	Xinyang 10	Henan	46	Mengshan 9	Sichuan
3	Huangjincha 2	Hunan	25	Baijin Jian	Unknown	47	Zifeng	Unknown
4	Ziyan	Sichuan	26	Bayu Tezao	Chongqing	48	Shuijing Nai Bai	Zhejiang
5	Mingxuan 213	Sichuan	27	Echa 10	Hubei	49	Fu’an Dabai Cha	Fujian
6	Jinyun 2	Zhejiang	28	Yujinxiang	Guangdong	50	Xiangfei Cui	Hunan
7	Jiu 20	Anhui	29	Mingshan Baihao 131	Sichuan	51	Xiaoxiang Hong 21-3	Hunan
8	Qihong 1	Anhui	30	Aolu	Japan	52	Guilv 1	Guangxi
9	Raoshan Xiulu	Henan	31	Wuniuzao	Zhejiang	53	Mingke 1	Fujian
10	Jin Mudan	Fujian	32	Gehe Cha	Fujian	54	Sanhua 1957	Sichuan
11	Yangshulin 781	Anhui	33	Zhonghuang 1	Zhejiang	55	Bai Yuxian	Zhejiang
12	Zhongbai 1	Zhejiang	34	Guicha 2	Guangxi	56	Zhongcha 102	Zhejiang
13	Ganlu 8	Sichuan	35	Bixiangzao	Hunan	57	Anji Baicha	Zhejiang
14	Dajiangzhong	Yunnan	36	Emei Wenchun	Sichuan	58	Tezao Huang	Unknown
15	Xishancha 1	Guangxi	37	Zhuye Qi	Hunan	59	Meizhan	Fujian
16	Jianbo Huang 13	Hubei	38	Yujinxiang	Zhejiang	60	Zhongcha 111	Zhejiang
17	Xiaoqingmiao	Unknown	39	Hongqi 1	Anhui	61	Fuding Dahao	Fujian
18	Chuancha 3	Sichuan	40	Xishancha 8	Guangxi	62	Cuifeng	Zhejiang
19	Guicha 1	Guangxi	41	Pingyang Tezaocha	Zhejiang	63	Maotouzhong	Zhejiang
20	Chungui	Fujian	42	Fuyun No.4	Fujian	64	Zhongcha 302	Zhejiang
21	Xiangguliao Baihao	Zhejiang	43	Xiangshan Zao 1	Henan	65	Shuchazao	Anhui
22	Fuzao 1	Anhui	44	Chuanmu 217	Sichuan			

## Data Availability

Additional data can be obtained by contacting the corresponding authors of the article.

## Supplementary Materials

The following supporting information can be downloaded at: https://www.mdpi.com/article/10.3390/plants15091300/s1, Table S1: Full names and abbreviations of the tea germplasm accessions; Table S2: Contents of biochemical components in the tested tea germplasm samples; Table S3: Contents of water-soluble fluoride in the tested tea germplasm samples; Table S4: Contents of seven mineral elements in representative tea germplasm samples.

## Abbreviations

The following abbreviations are used in this manuscript:

EGC	Epigallocatechin
EGCG	Epigallocatechin gallate
WSF	Water-soluble fluoride
PCA	Principal component analysis
HCA	Hierarchical cluster analysis
TP	Total tea polyphenol
EC	Epicatechin
C	Eatechin
ECG	Epicatechin gallate
CG	Catechin gallate
WE	Water extract
FAA	Free amino acids
CAF	Caffeine
K	Potassium
B	Boron
Zn	Zinc
Ca	Calcium
Mn	Manganese
Al	Aluminum
Mg	Magnesium
TC	Total catechins
NETC	Non-esterified catechins
ETC	Esterified catechins
CQI	Catechin quality index
SD	Standard deviation
CV	Coefficient of variation
DW	Dry weight
